# Factors Impacting Treatment Decision Making in Older Adults With Indolent Non-Hodgkin Lymphoma

**DOI:** 10.1093/geroni/igab046.1985

**Published:** 2021-12-17

**Authors:** Kelly Trevino, Peter Martin, John Leonard

**Affiliations:** 1 Memorial Sloan Kettering Cancer Center, New York, New York, United States; 2 Weill Cornell Medicine, New York, New York, United States

## Abstract

Indolent non-Hodgkin lymphomas (NHL) are among the most common lymphomas and up to two-thirds of diagnoses are made in older adults (age≥65 years). Initial treatment options include cancer-directed therapy or active monitoring by the oncologist for disease progression. Despite the disparate nature of these treatments, the factors impacting older adults’ treatment decisions are unknown. This study examines the reasons older adults chose their initial treatment, factors influencing this decision, shared decision-making preferences, and differences in these factors relative to younger adults (age<65 years). Adult patients (≥21 years) with a new diagnosis of indolent NHL in the past six months completed electronic self-report measures. The final sample consisted of 86 patients; 43.0% (n=37) were older adults. Over two-thirds of older adults (n=25, 67.6%) were being monitored by their oncologist with no age differences in current treatment (p=.55). Most older adults chose their treatment plan to “maximize my long-term health” (n=24, 64.9%) which did not differ from younger adults (p=.77). The primary factors impacting older adults’ treatment decisions were their doctor’s recommendation (M=3.92, SD=.28, Range=0-4) and their personal preference (M=2.88, SD=1.68, Range=0-4). Factors impacting treatment decisions did not differ by age (all p’s>.05). Most older adults (n=25, 69.4%) expressed a preference for shared decision-making with their oncologist which did not differ from younger adults (p=.17). Treatment planning for older adults should consider long-term health, consistent with older adults’ values. Older adults may view treatment decision-making similarly to younger adults; assumptions about patients’ values and decision-making preferences based on age are likely inappropriate.

